# Resilience in Tunisian women: The critical role of social support

**DOI:** 10.1192/j.eurpsy.2025.1615

**Published:** 2025-08-26

**Authors:** H. Mhiri, I. Chaari, I. Mannoubi, N. Boussaid, F. Charfeddine, L. Aribi, N. Messedi, J. Aloulou

**Affiliations:** 1 Psychiatric department “B”, Hedi Chaker University Hospital, Sfax, Tunisia

## Abstract

**Introduction:**

Resilience is the capacity to bounce back from adversity, and this concept has been extensively studied across various populations, especially those facing significant stress. In this context, Tunisian women have recently encountered multiple challenges related to balancing professional and family responsibilities, which require them to demonstrate strong resilience. Recent literature has highlighted a strong link between social support and resilience.

**Objectives:**

This study aims to explore the impact of social support on the psychological resilience of Tunisian women.

**Methods:**

This cross-sectional study surveyed Tunisian women aged 18 and above using an online questionnaire between June and August 2024. Familial and professional characteristics were collected through a structured survey. Social support was measured using the Multidimensional Scale of Perceived Social Support (MSPSS), and psychological resilience was assessed with the 25-item Connor-Davidson Resilience Scale (CD-RISC 25).

**Results:**

Data from 695 Tunisian women (mean age = 36.72 ± 12.23 years) revealed diverse backgrounds: 24.7% were students, 35.3% worked in the public sector, 21.2% were self-employed, and 14.7% were unemployed. In terms of marital status, 42.9% were single, 49.2% married, and 5.6% divorced, with 8.5% living alone, 75.7% in nuclear families, 10.4% in extended families, and 5.5% in shared housing; additionally, 50.6% had children. The average resilience score was 68.26 ± 14.09, with 26.3% exhibiting low resilience. Social support scores were as follows: familial support (20.06 ± 6.38), friend support (18.17 ± 6.47), significant other support (21.2 ± 6.22), and overall social support (59.42 ± 15.26). Significant associations were found between resilience and marital status (p = 0.019), with married women showing lower rates of low resilience (21.3%) than single women (31.9%). Women with children also demonstrated higher resilience (p = 0.002), while employment status had a notable impact (p = 0.005), with low resilience rates highest among unemployed women (36.3%) and lowest among public sector workers (19.6%). Resilience positively correlated with social support across all sources: familial (p < 0.001, r = 0.269), friend (p < 0.001, r = 0.260), significant other (p < 0.001, r = 0.289), and overall social support (p < 0.001, r = 0.338).

**Conclusions:**

Our findings underscore the crucial role of social support and family connections in enhancing the psychological resilience of Tunisian women. Therefore, targeted social interventions are needed to support women experiencing loneliness or lacking social networks, aiming to strengthen their resilience and overall well-being.

**Disclosure of Interest:**

None Declared

